# Progress in Bacteriology

**Published:** 1902-05-03

**Authors:** 


### PROGRESS IN BACTERIOLOGY.

**Malaria.**—Ray Lankester[1] proposes the following terminology for the various stages of the malaria parasite. The malarial germ as it leaves the anopheles and enters the blood-stream is called an exotospore. These, as soon as they enter a red blood-cell, become amœbulæ, and after going through various stages of development divide up into small segments. Enhæmospores, which may either enter fresh blood-corpuscles or may, in the stomach of a mosquito, become converted into male and female crescents. The female crescent becomes an egg-cell and the male crescent a sperm-cell, and these fusing form a zygote. The zygote undergoes a change in shape and becomes a vermicule, which, burrowing through the stomach wall, becomes encysted in the blood sinuses and forms a spore cyst. This cyst grows rapidly in size, and, by multiplication of its cells, forms a number of spore mother-cells, which finally give rise to a crop of filiform spores (exotospores), which, on the rupture of the cyst, enter the blood of the mosquito and are carried to the ducts of the salivary gland, from whence they may be injected into the human body.

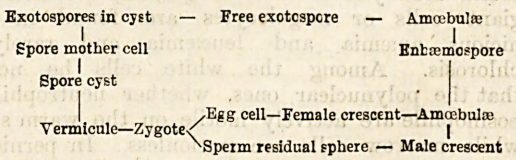

The rôle of protozoa in the causation of disease is discussed by McWeeney.[2] The order consists of four classes: rhizopoda, sporozoa, flagellata, and infusuria. Of the rhizopoda, the amœba coli is parasitic in man, and either alone or with the assistance of bacteria excites the condition known as tropical dysentery. Amongst the sporozoa the coccidia were pathogenic to animals and possibly to man, and their development in many ways closely resembles that of the malaria parasites. They infect the cells of the bile duct and intestine, split up into numerous sickle-shaped segments (merozoites), which escape and infect other cells. Later, a bisexual development takes place and male and female microgametes are formed, which fuse and form a "sporent," which escapes with the fæces, is taken in by another rabbit, develops two sickle-shaped germs and starts the process afresh. This sexual process closely resembles that of the malaria parasites, with the exception that no cold-blooded host, such as the mosquito, is necessary. Of the flagellata the Trypanosoma Lewisii was often found in the blood of apparently healthy wild rats, and the Trypanosoma Brucii excited the diseases known as tsetse fly and surra in cattle. Of uncertain classification were Piroplasma begemenum, the exciting cause of Texas fever; the supposed parasite of vaccinia described by Guarnieri; and the forms found in malignant growths by Sanfelice. Leishman [3] suggests the following modification of Romanowsky's stain for the malaria parasite. A solid double-staining precipitate is obtained by mixing together the following two solutions: (*a*) A 1-in-1,000 solution of methylene blue with 0·5 per cent. of sodium carbonate; (*b*) A 1-in-1,000 solution of eosin, extra B, in distilled water. This precipitate is washed in distilled water, dried and powdered, and dissolved in the proportion of 0·15 per cent. in pure methyl alcohol. This alcohol fixes the blood film, and the staining procedure is as follows: Allow the stain to remain on the film for half a minute, then add double the quantity of distilled water, and allow the whole to remain for five or ten minutes; wash for two minutes in distilled water, dry and mount. The red cells have a pink or green tint, the bodies of the malarial parasites are blue, their chromatin ruby red. Polynuclear leucocytes have a red nuclear network, colourless extra nuclear protoplasm, and red eosinophil granules.

[1] Brit. Med. Jour., March 15, 1902. [2] Lancet, Nov. 16, 1901. [3] Brit. Med. Jour., Sept. 21, 1901.

